# CXCL1 promotes colon cancer progression through activation of NF-κB/P300 signaling pathway

**DOI:** 10.1186/s13062-022-00348-4

**Published:** 2022-11-25

**Authors:** Changhua Zhuo, Qiang Ruan, Xiangqian Zhao, Yangkun Shen, Ruirong Lin

**Affiliations:** 1 Department of Gastrointestinal Surgical Oncology, Clinical Oncology School of Fujian Medical University, Fujian Cancer Hospital, Fuzhou, Fujian 350014 People’s Republic of China; 2 Fuzhou University, College of Chemistry, Fuzhou, 350108 People’s Republic of China; 3 Fujian Key Laboratory of Translational Cancer Medicine and Fujian Provincial Key Laboratory of Tumor Biotherapy, Fuzhou, Fujian 350014 People’s Republic of China; 4 Fujian Normal University Qishan Campus, College of Life Science, Biomedical Research Center of South China, Fuzhou, 350117 People’s Republic of China

**Keywords:** CXCL1, Colon cancer, NF-κB, P300, C646

## Abstract

**Background:**

The upregulated expression of CXCL1 has been validated in colorectal cancer patients. As a potential biotherapeutic target for colorectal cancer, the mechanism by which CXCL1 affects the development of colorectal cancer is not clear.

**Methods:**

Expression data of CXCL1 in colorectal cancer were obtained from the GEO database and verified using the GEPIA database and the TIMER 2.0 database. Knockout and overexpression of CXCL1 in colorectal cancer cells by CRISPR/Cas and "Sleeping Beauty" transposon-mediated gene editing techniques. Cell biological function was demonstrated by CCK-8, transwell chamber and Colony formation assay. RT-qPCR and Western Blot assays measured RNA and protein expression. Protein localization and expression were measured by immunohistochemistry and immunofluorescence.

**Results:**

Bioinformatics analysis showed significant overexpression of CXCL1 in the colorectal cancer tissues compared to normal human tissues, and identified CXCL1 as a potential therapeutic target for colorectal cancer. We demonstrate that CXCL1 promotes the proliferation and migration of colon cancer cells and has a facilitative effect on tumor angiogenesis. Furthermore, CXCL1 elevation promoted the migration of M2-tumor associated macrophages (TAMs) while disrupting the aggregation of CD4+ and CD8+ T cells at tumor sites. Mechanistic studies suggested that CXCL1 activates the NF-κB pathway. In the in vivo colon cancer transplantation tumor model, treatment with the P300 inhibitor C646 significantly inhibited the growth of CXCL1-overexpressing colon cancer.

**Conclusion:**

CXCL1 promotes colon cancer development through activation of NF-κB/P300, and that CXCL1-based therapy is a potential novel strategy to prevent colon cancer development.

## Introduction

Colorectal cancer is one of the most common malignancies. Globally, there were approximately 1.15 million new cases of colorectal cancer in 2020, accounting for 6.0% of all new cancer cases. In the same year, there were approximately 580,000 deaths due to colorectal cancer, accounting for 5.8% of all cancer-related deaths [1]. Colorectal cancer is the third most common and fatal tumor among both males and females in the United States. According to the latest epidemiological survey, there were an estimated 149,500 new cases and 52,980 deaths in 2021 [2]. The main cause of colon cancer-related mortality is resistance to the current standard chemotherapy regimens, leading to a high incidence of metastatic recurrence [3]. Therefore, identifying effective therapeutic targets for colorectal cancer and improving the sensitivity of chemotherapy are key imperatives to increase the survival rate of patients.

The Nuclear Factor “kappa-light-chain-enhancer” of activated B-cells (NF-κB) is an important immune defense transcription factor that mediates cytoplasmic/nuclear signaling pathways [4] and regulates the gene expression of various cytokines and cytokine receptors and adhesion molecules in the inflammatory nuclear immune response [5]. It is also associated with apoptotic pathways, cell proliferation and differentiation, and resistance to chemotherapy/radiotherapy in tumors [6, 7]. Therefore, targeting the NF-κB pathway may be an effective means of preventing cancer progression and improving chemotherapy sensitivity.

The chemokine C-X-C motif ligand 1 (CXCL1), also known as GROα, belongs to the CXC chemokine family and is secreted by macrophages, neutrophils, and epithelial cells. CXCL1 signaling is through binding to the G protein-coupled chemokine receptor CXCR2 [8], which is predominantly expressed on myeloid cell populations (neutrophils, monocytes, and macrophages) [9]. This receptor directs the efflux of myeloid cells from the bone marrow and their migration to tumor sites with high CXCL1 expression, where they promote tumor immune escape by inhibiting the proliferation, activation, and motility of effector T cells [10, 11] and stimulating the expansion of Treg [12].

Studies have demonstrated high expression of CXCL1 in a variety of cancers and its association with cancer progression and inflammation. For example, several studies have found elevated levels of CXCL1 in plasma, serum, ascitic fluid, and tumor tissue of patients with ovarian cancer [13, 14]. High CXCL1 expression showed a significant association with lymph node metastasis and poor overall survival in patients with breast cancer [15]. In our previous study, we found high expression of CXCL1 in colorectal cancer and its close correlation with the clinicopathological features; in addition, CXCL1 overexpression was closely associated with tumor diameter, stage, degree of infiltration, and lymph node metastasis [16]. Wang et al. suggested that CXCL1 may enhance the metastasis of colorectal cancer by interacting with CXCR2 [17]. However, the specific mechanism of action of CXCL1 in colorectal cancer is not well characterized.

The current study was designed to study the molecular mechanism by which CXCL1 promotes the progression of colorectal cancer. We hypothesized that CXCL1 promotes the development of colon cancer through the NF-κB/P300 pathway. We first verified the differential expression of CXCL1 in colorectal cancer, and found significantly higher expression of CXCL1 in colorectal cancer compared to that in normal tissues. Knockout of *CXCL1* in colon cancer cells significantly inhibited the growth of colon cancer, while overexpression of CXCL1 showed the opposite result. In the mouse model, treatment with P300 inhibitor, C646, led to significant inhibition of tumor growth after overexpression of CXCL1. Bioinformatics analysis using the TCGA database highlighted the clinical relevance of our findings. These data not only suggest that CXCL1 may be a potential therapeutic biomarker of colorectal cancer, but also reveal the potential mechanism by which CXCL1 promotes the progression of colorectal cancer. Our results may help inform novel strategies for targeted therapy of colorectal cancer.

## Material and methods

### Data sources and descriptions

GSE113513, GSE25070, GSE41328, and GSE156355 were the five mRNA expression datasets (including colorectal tumors and normal tissues) obtained from the GEO database (https://www.ncbi.nlm.nih.gov/geo/).

The sequencing dataset and patient parameters for colorectal cancer were obtained from TCGA, containing 698 samples of 51 normal and 647 tumor types. The expression of CXCL1 in tumors and paracancerous tissues of colorectal cancer patients were compared to evaluate its diagnostic value. The staging correlation of colorectal cancer phenotypes with high and low CXCL1 expression was analyzed according to the median expression level.

### Identification of the CXCL1 expression profile

The raw expression data from the above five GEO datasets were preprocessed into expression matrices using R software and Microsoft Excel 2019. One of the R commands, NormizeBetween Arrays, was executed to normalize the raw expression data. Differentially expressed genes were analyzed by running the LIMMA package, which is a collection of R commands. By using ImageGP (http://www.ehbio.com/ImageGP/index.php/Home/Index/index.html), a web tool for visualizing clustering of multivariate data, the common differentially expressed genes (DEGs) of the above five datasets were identified and displayed in a Venn diagram.

### Expression of CXCL1 in colorectal cancer and normal tissues

Human Protein Atlas (HPA: https://www.proteinatlas.org/) was used to reveal the distribution of CXCL1 in normal human tissues. Gene Expression Profiling Interactive Analysis (GEPIA: http://gepia.cancer-pku.cn/index.html) and Tumor Immune Estimation Resource (TIMER 2.0: http://timer.cistrome.org/) were used to explore the distribution of CXCL1 in colorectal cancer and normal colon tissues.

### Cell lines and culture conditions

Mouse colon cancer cell line MC38 was obtained from the Shanghai Institute of Digestive Surgery (Shanghai, China). The MC38^CXCL1−/−^ cell line was generated using the CRISPR/Cas 9 technique (see below for details); the MC38^CXCL1+/+^ cell line was obtained by transducing CXCL1 to MC38 using the “Sleeping Beauty” transposon (see below for details). Cell lines were cultured in high-sugar DMEM medium containing 1% penicillin/streptomycin and 10% fetal bovine serum at 37 °C with 5% CO_2_ humidification.

### RNA extraction and real-time fluorescence quantitative PCR

Total RNA was extracted from colon cancer lines using Trizol reagent (Magen, R4801-02, Shanghai, China) and 1 µg of total RNA was reverse transcribed using the PrimeScript RT reagent kit (Vazyme, Nanjing, China) to detect relevant mRNAs. Real-time fluorescence quantitative PCR was performed using SYBR Premix Ex Taq II (Vazyme, Nanjing, China) at 95 °C for 30 s, followed by 40 cycles of 95 °C for 5 s, 55 °C for 30 s, and 72 °C for 30 s. The primer sequences used for PCR are shown in Table 1. Changes in relative expression of different samples were calculated by the ABI Q6 Fast real-time PCR system (Applied Biosystems, Foster City, CA, USA) using the 2^−∆∆CT^ method. GAPDH was used as an internal reference gene.

**Table 1:** Primer sequences used for qRT-PCR analyses

Gene	Primer	Sequence
Mouse GAPDH	m-GAPDH-F	CCAGAGCTGAACGGGAAGCTCAC
	m-GAPDH-R	CCATGTAGGCCATGAGGTCCACC
Mouse CXCL1	m-CXCL1-F	AGCTGCGCTGTCAGTGCC
	mCXCL1-R	CAAGCCTCGCGACCATTC
Mouse IL-6	m-IL6-F	CTCCCAACAGACCTGTCTATAC
	m-IL6-R	CCATTGCACAACTCTTTTCTCA
Mouse IL-1β	m-IL1β-F	AAATGCCACCTTTTGACAGTGA
	m-IL1β-R	GGTTTGGAAGCAGCCCTTCA
Mouse TNF-α	m-TNFα-F	GATCGGTCCCCAAAGGGATG
	m-TNFα-R	CCACTTGGTGGTTTGTGAGTG
Mouse P300	m-P300-F	GCCAACATTGGAGGCACTTT
	m-P300-R	GCCAACATTGGAGGCACTTT

### CRISPR/Cas9-mediated gene knockout

The designed gRNA oligonucleotides were annealed and cloned into the vector pX459 (the primer sequences: m-CXCL1-sg-F: GCTGCTGGCCACCAGCCGCC; m-CXCL1-sg-R: GGCGGCTGGTGGCCAGCAGC). To knock out the target gene, we introduced the pX459 eukaryotic expression vector carrying the corresponding gRNA into MC38 cells using Lipofectamine 2000 (Thermo Fisher Scientific, Waltham, MA, USA) and screened with 1 µg/mL of puromycin for 3 days after 1 week. Subsequently, the surviving cells were transferred to fresh culture medium without puromycin for 2 days. Plates were spread at an ultra-low density to ensure clone formation from individual cells, and then clones were selected and expanded for gene knockout validation by sequencing and Western Blot assay of the target genomic region.

### “Sleeping Beauty” transposon-mediated gene transfer

CXCL1 was constructed into vector PT2/SVNeo to establish PT2/SVNeo-CXCL1 recombinant transposon vector. To overexpress the target gene, we mixed the recombinant transposon vector and SB100 in a homogeneous ratio of 1:2, introduced into MC38 cells using Lipofectamine 2000 (Thermo Fisher Scientific, Waltham, MA, USA), and spread the plate at ultra-low density after 48 h to ensure clone formation from individual cells. Then the clones were selected and expanded. The relative mRNA expression of the target gene was verified by real-time fluorescence quantitative PCR.

### Western Blot analysis

Different cells were washed separately with ice-cold PBS, gently scraped with a cell spatula, and lysed using radioimmunoprecipitation assay (RIPA) cell lysis buffer (Beyotime, P0013B, Shanghai, China). Quantification was performed using the BCA protein concentration assay kit (Beyotime, P0012S, Shanghai, China). 60 µg of total protein was passed through 12.5% SDS polyacrylamide gels, and then transferred to PVDF membranes (Bio-Rad, Hercules, CA). The membranes were closed in 5% BSA for 2 h and then incubated overnight with the corresponding primary antibodies at 4 °C, followed by incubation with the corresponding IRDye 800CW or 680 LT secondary antibodies (1:10,000, Abcam, China) in dark for 1 h at room temperature. The fluorescence signals of membranes were detected using Odyssey CLx Western blot detection system (Westburg, Leusden, the Netherlands). The images were analyzed using ImageJ 1.43 software. The expression of β-actin was used as an endogenous control. Information about all antibodies is listed in Table 2.

**Table 2:** Antibodies used for Western Blot, Immunohistochemistry, and Immunofluorescence

Antibodies	Source	identifier
Anti-beta Actin antibody	Abcam	Cat#ab8227
GRO alpha Rabbit pAb	ABclonal	Cat#A5802
CXCR2 Rabbit pAb	ABclonal	Cat#A3301
Phospho-IκBα Rabbit mAb	Cell Signaling Technology	Cat#2859
IκBα Rabbit mAb	Cell Signaling Technology	Cat#4812
Phospho-NF-κB p65 Rabbit mAb	Cell Signaling Technology	Cat#3033
NF-κB p65 Rabbit mAb	Cell Signaling Technology	Cat#8242
Anti-CD31 antibody	Abcam	Cat#ab9498
Anti-CD4 antibody	Abcam	Cat#ab183685
Anti-CD8 antibody	Abcam	Cat#ab217344
Anti-CD163 antibody	Abcam	Cat#ab182422
Donkey Anti-Rabbit IgG H&L	Abcam	Cat#ab150075
DAPI	ZSGB-BIO	Cat#ZLI-9557

### Cell proliferation assay

MC38 WT, MC38^CXCL1−/−^, and MC38^CXCL1+/+^ cells were inoculated onto 96-well plates at a density of 5 × 10^3^ cells/well, respectively. After apposition of cell, cell viability was assayed using a cell counting kit (CKK8, Beyotime, China) according to the manufacturer’s instructions to ensure consistent numbers of various cell inoculations. Cell proliferation was subsequently assayed every 24 h for a total of 4 times.

### Transwell migration assay

Transwell chambers were used to assess cell migration. A culture medium containing 10% fetal bovine serum was added to the lower chamber. 1 × 10^5^ colon cancer cells (MC38 WT, MC38^CXCL1−/−^, and MC38^CXCL1+/+^) were suspended in serum-free culture medium and inoculated in the upper chamber, respectively. After 24 h incubation, the colon cancer cells were removed from the upper chamber with a cotton swab. The cancer cells that penetrated and adhered to the bottom of the filter membrane were fixed with 4% paraformaldehyde in PBS for 15 min, then stained with 0.5% crystal violet for 20 min, and then imaged under 20 × magnification. For statistical analysis, the number of invading cells was calculated from three independent experiments, with an average from four image fields per experiment. The colonies were observed under the microscope.

### Colony formation assay

The groups of cells (MC38 WT, MC38^CXCL1−/−^, MC38^CXCL1+/+^) were inoculated at a density of 6 × 10^2^ cells/well in six-well plates. After 10 days, cells were washed 3 times with PBS and fixed with 4% paraformaldehyde for 30 min, followed by staining with 0.5% crystalline violet staining solution.

### ELISA analysis

The culture supernatant derived from cell medium was collected and the concentration of the CXCL1 secretory protein levels was determined using mouse CXCL1 ELISA kit (Solarbio, Beijing, China) according to the manufacturer's method. Absorbance was measured at 450 nm.

### Animal experiments

All animal experiments were conducted according to the *Guide for the Care and Use of Laboratory Animals* approved by the Fujian Provincial Office for Managing Laboratory Animals and were guided by the Animal Care and Use Committee of Fujian Medical University. Six-week-old C57BL/6 male mice were purchased from the Shanghai SLAC Laboratory Animal Co. (Shanghai, China), and housed at a density of five to six mice per cage in a controlled environment (12 h daylight/dark schedule) and provided ad libitum access to food and water. MC38, MC38^CXCL1−/−^, and MC38^CXCL1+/+^ cells were suspended in PBS (1 × 10^6^ cells, 100 µL) and injected subcutaneously into the right flank of mice to establish mouse colon cancer tumor models.

Mice (MC38, MC38^CXCL1−/−^, and MC38^CXCL1+/+^) were divided into NC group (n = 5) and C646 group (n = 5). Mice in the c646 groups were administered intraperitoneal injection of c646 every 3 days (MCE, 30 nmol/g) for 2 weeks. Mice in the NC groups were administered the same volume of PBS placebo. The tumors of mice were measured every 3 days with vernier calipers and the tumor volume was calculated using the following formula: V = (length) × (width)^2^/2.

### Immunohistochemistry

Tumor specimens were fixed in 10% neutral buffered formalin for 24 h, followed by standard tissue processing and embedding. Sections of paraffin-embedded tumor specimens were cut at 4 µm and dried overnight at 37 °C. Sections were then dewaxed twice in xylene for 10 min each and rehydrated by passage through a graded ethanol series. Endogenous peroxidase was inactivated using 3% hydrogen peroxide for 30 min at room temperature. The slides were heated in citrate buffer for antigen repair. The slides were then incubated overnight with primary antibodies in a humid chamber at 4 °C. DAB detection system (ZSGB-BIO, Beijing, China) was used as a chromogenic agent according to the manufacturer’s instructions. Finally, sections were re-stained with Mayer’s hematoxylin, dehydrated, and sealed. Digital images of the stained sections were acquired with Axiolmage2 ortho-fluorescence phase contrast microscope (Zeiss, Oberkochen, Germany). Information about all antibodies is listed in Table 2.

### Immunofluorescence

The first half of the immunofluorescence step for tumor tissues was similar to immunohistochemistry. Primary antibodies were removed and washed three times with PBS for 10 min each, and incubated with secondary fluorescent labeling antibodies at room temperature for 2 h. Finally samples were incubated with 4,6-diamidino-2-phenylindole for nuclear staining. Tumor tissues were imaged using an Axiolmage2 ortho-fluorescence phase contrast microscope (Zeiss, Oberkochen, Germany) for section acquisition.

Primary antibodies were diluted at 1:1000 using immunostaining primary antibody dilution (Beyotime, P0103, Shanghai, China) and fluorescent secondary antibody was diluted at 1:1000 using immunostaining secondary antibody dilution (Beyotime, P0108, Shanghai, China).

### Statistical analysis

Data were expressed as mean ± standard error of the mean (SEM). Statistical analysis was performed using GraphPad Prism 8.0 software. Between-group differences were assessed using Student’s *t* test or one-way analysis of variance (ANOVA). For all analyses, *P* values < 0.05 were considered indicative of statistical significance. *P < 0.05, **P < 0.01, ***P < 0.001, ****P < 0.0001.

## Results

### CXCL1 identified as an upregulated gene in GEO datasets

**Fig. 1:**
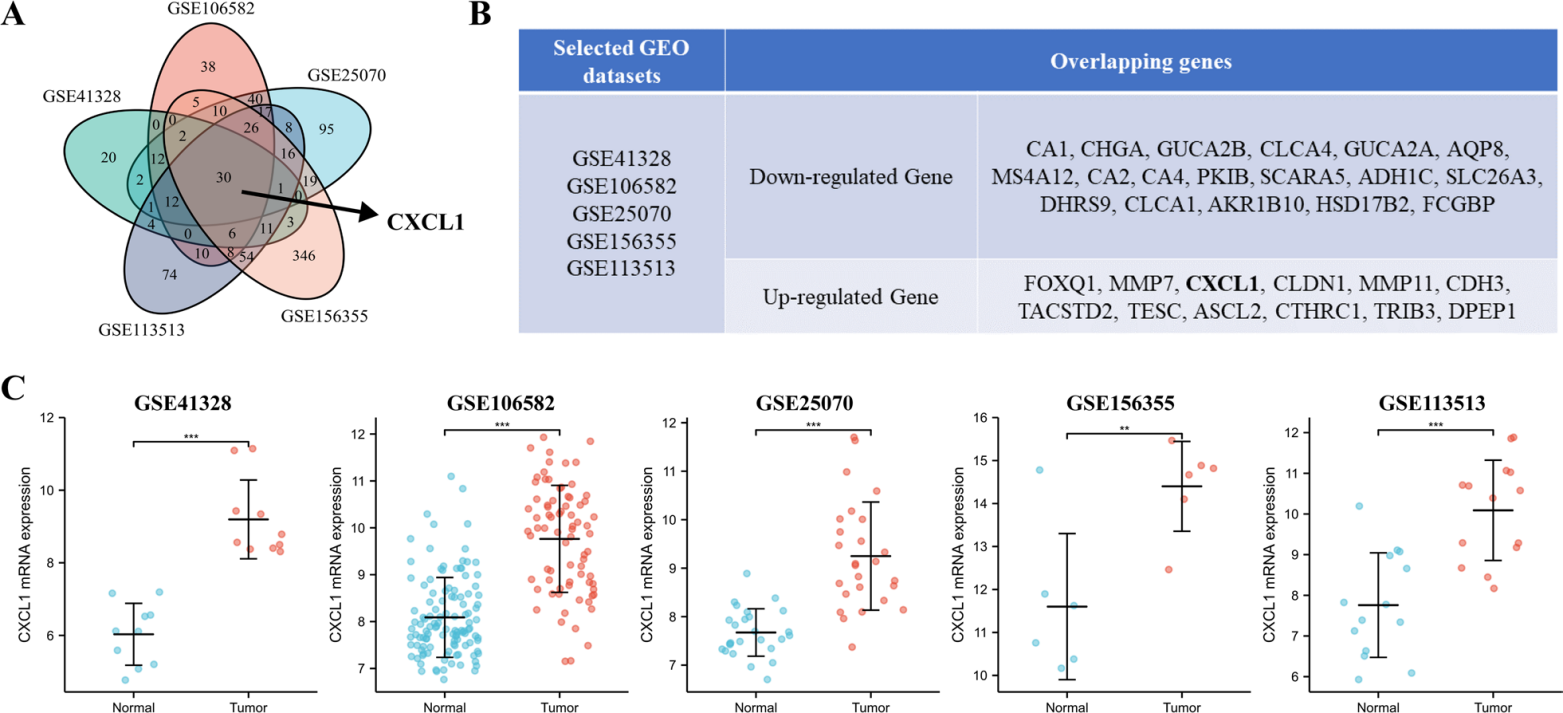
Analysis of the CXCL1 expression profile in the five GEO databases (GSE41328, GSE106582, GSE25070, GSE156355, and GSE113513). **a** Venn diagram showing the commonly differentially expressed genes in colorectal cancer; **b** total symbols of the overlapping down-regulated and up-regulated genes; **c** *CXCL1* mRNA expression between normal tissues and colorectal cancer tissues. **P < 0.01, ***P < 0.001

To explore the key genes in colorectal cancer, we simultaneously performed differential gene expression analysis in the five selected GEO datasets. The results showed 30 differentially expressed genes that were common to these five datasets (Fig. 1a, b). Among these 30 overlapping genes, 18 genes were down-regulated genes and 12 genes were up-regulated genes, and *CXCL1* was one of the 12 up-regulated genes. The other 11 up-regulated genes, such as *FOXQ1, MMP7, MMP11*, and*CDH3*, have been reported in previous cancer-related studies [18–22]. The function and mechanism of action of *CXCL1* have seldom been researched in the context of colorectal cancer. Therefore, we selected *CXCL1* as the main object of interest. As shown in Fig. 1c, *CXCL1* expression was significantly higher in colorectal cancer tissues than in the paratumoral normal tissues.

**Fig. 2:**
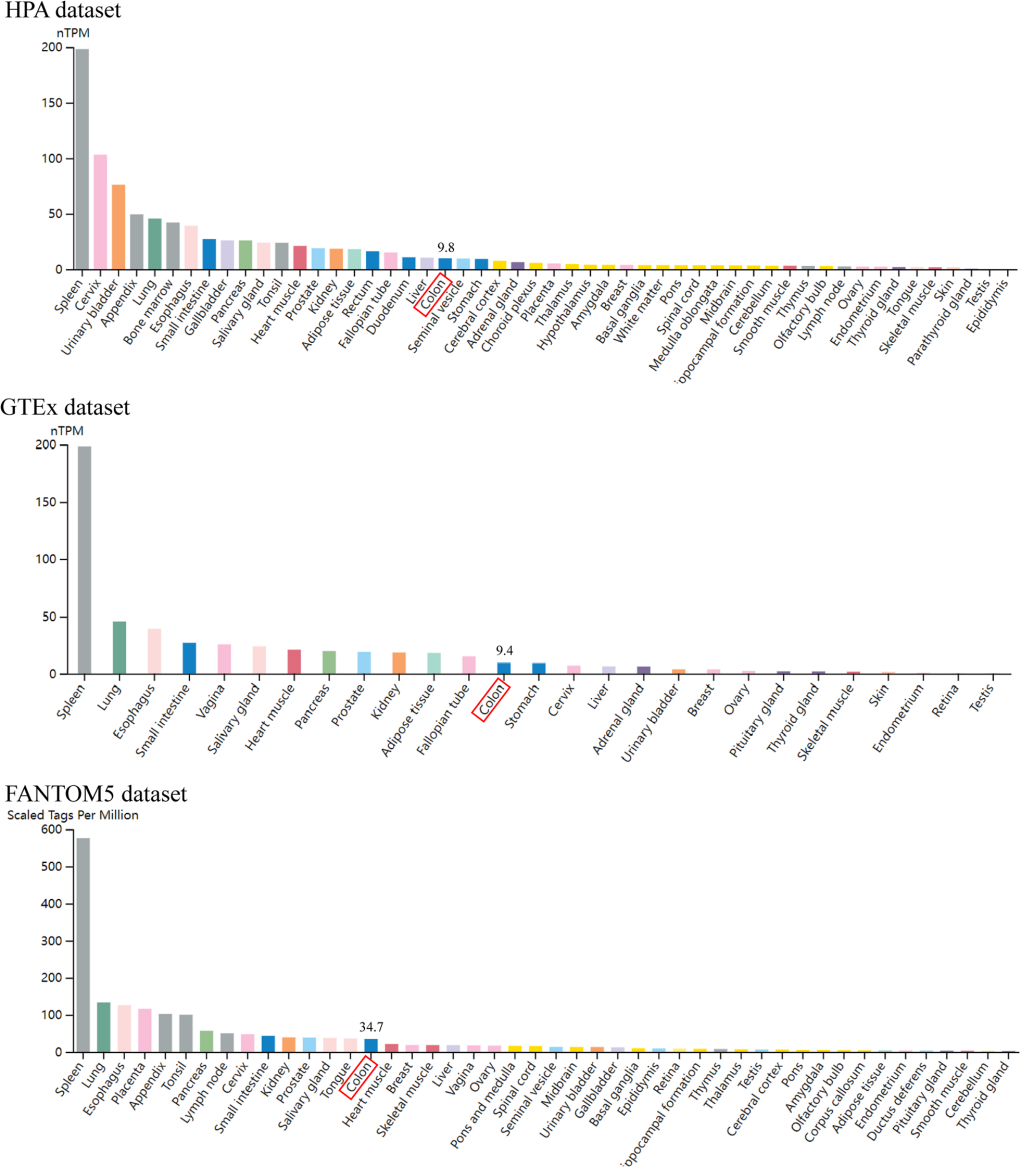
Expression level of CXCL1 in normal human tissues. *HPA* Human Protein Atlas, *GTEx* Genotype-Tissue Expression, *FANTOM5* Functional Annotation of Mammalian Genomes 5

**Fig. 3:**
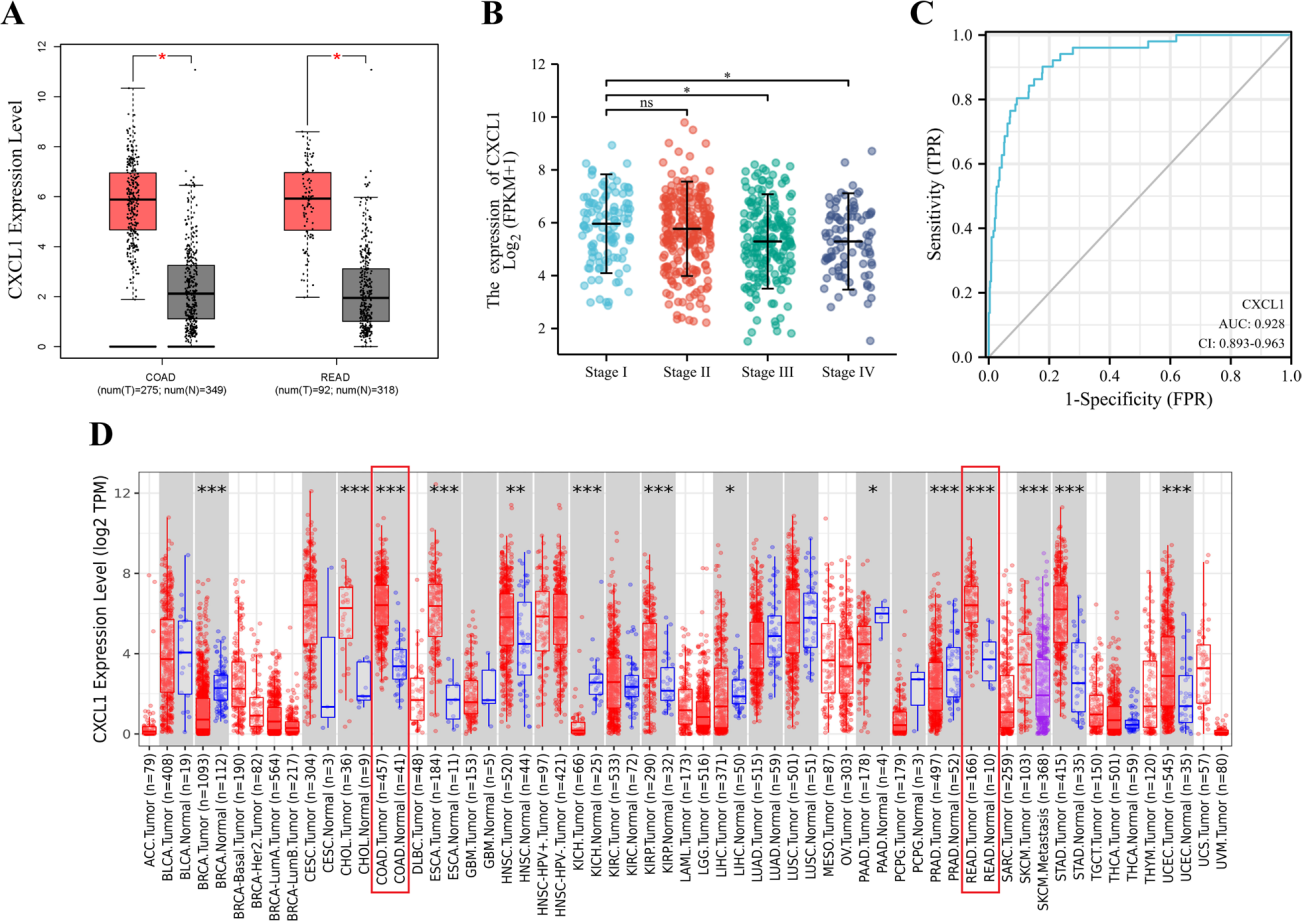
**a** *CXCL1* expression profile in colorectal cancer based on data from the GEPIA database. **b** Correlation of *CXCL1* expression with clinical characteristics of patients with colorectal cancer; **c** the ROC curves of *CXCL1* gene mRNA expression in tumor tissues and paratumoral normal colorectal tissues of TCGA cohort. **d** *CXCL1* expression profile in colorectal cancer based on data from TIMER 2.0 database. *ns* no significant, *P < 0.05, **P < 0.01, ***P < 0.001

To further validate CXCL1 expression, we first analyzed CXCL1 expression in human normal tissues. CXCL1 expression in human normal tissues was obtained from Human Protein Atlas, and the data were based on HPA RNA-seq, Genotype-Tissue Expression (GTEx), and Functional Mammalian Genomes 5 dataset (FANTOM5) (Fig. 2). CXCL1 was found to be highly expressed in human normal colon tissues. Subsequently, we analyzed CXCL1 expression profiles in the GEPIA database and TIMER 2.0 database (Fig. 3a, d), and based on expression data in the databases, we determined that CXCL1 expression was significantly higher in human colorectal cancer tissues than in paratumoral normal tissues. These findings indicated the potential involvement of overexpression of CXCL1 in the genesis and progression of colorectal cancer.

### Colorectal cancer data analysis in TCGA database

To further investigate the effect of CXCL1 on colorectal cancer, we compared the expression of CXCL1 in colorectal cancer tissues and paratumoral normal tissues of patients with colorectal cancer using the TCGA database. In addition, we evaluated its diagnostic value, and staged colorectal cancer phenotypes with high and low CXCL1 expression according to the median expression level. A total of 644 colorectal cancer patients were included in this analysis, and RNA sequencing was performed on 647 tumor tissues and 51 paratumoral normal colorectal tissue specimens from these 644 patients. The analysis showed a decrease in *CXCL1* expression with the progression of tumor stage (Fig. 3b). Receiver operating characteristic (ROC) curve (Fig. 3c) showed that CXCL1 had a high accuracy for the diagnosis of colorectal cancer [area under the curve (AUC) (95% CI): 0.928 (0.893–0.963)]. The above analysis suggests that CXCL1 is pathologically and clinically associated with the development and progression of colorectal cancer.

### CXCL1 promotes the proliferation and migration of colon cancer cells

**Fig. 4:**
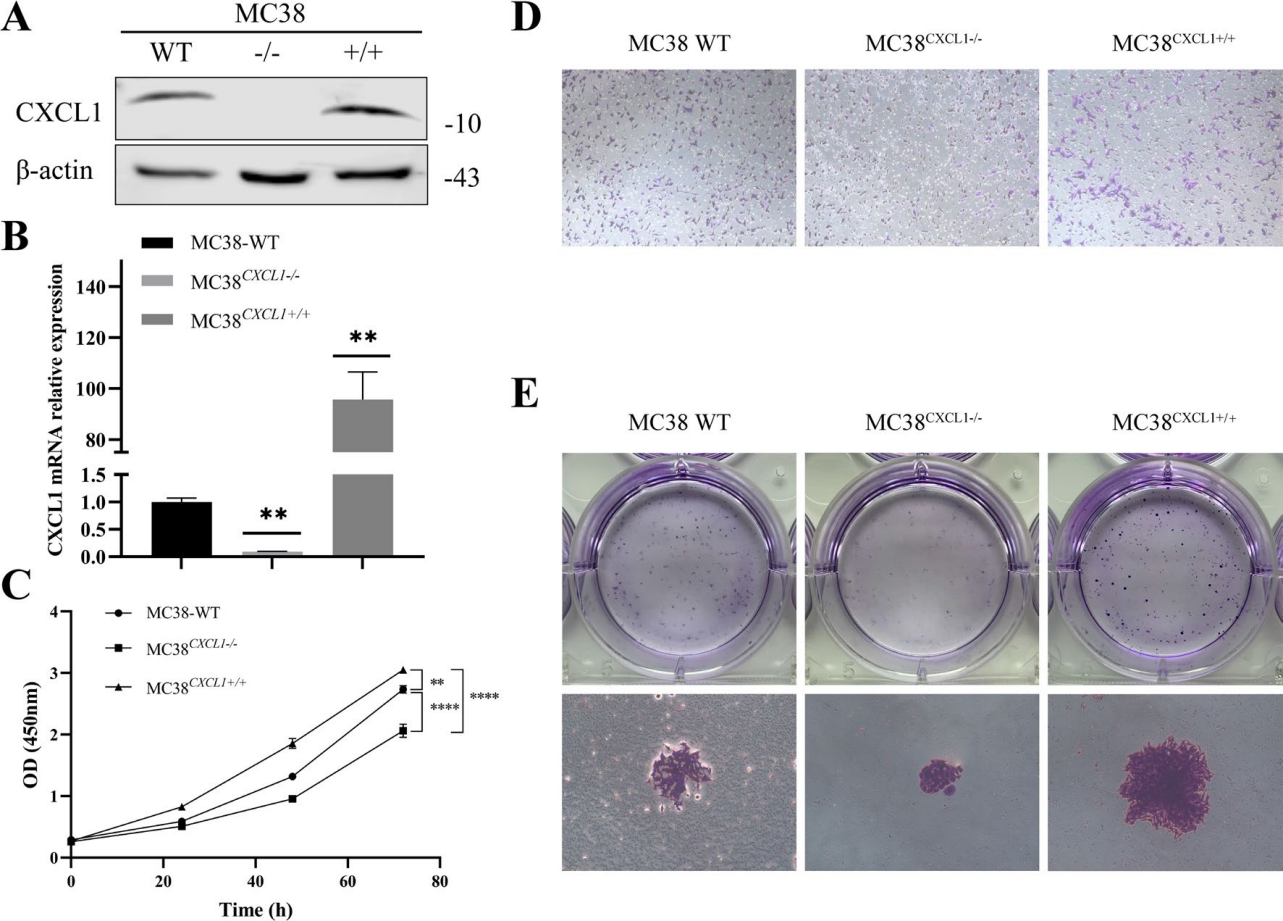
CXCL1 promotes colon cancer cell proliferation, migration, and invasion. **a** Western Blotting validation of CXCL1 knockout and overexpression efficiency in MC38 cells; **b** qPCR validation of CXCL1 overexpression efficiency in MC38 cells; **c** results of CCK8 assay showing a significant effect of CXCL1 on the proliferation of MC38 colon cancer cells. **d** Results of Transwell assay showing the stimulatory effect of CXCL1 on the migration and invasion ability of MC38 cells. **e** Cell colony formation ability assessed by plate clone formation assay. CXCL1 promoted the clonogenic ability in MC38 cells. **P < 0.01, ****P < 0.0001

In order to test the hypothesis that CXCL1 promotes colorectal cancer progression, we used CRISPR/Cas9 technology to knockout *CXCL1* in MC38 colon cancer cells to construct MC38^CXCL1−/−^ cell line; in addition, we also used “Sleeping Beauty” transposon to transduce CXCL1 into MC38 cells to construct MC38^CXCL1+/+^ cell line. The knockout and overexpression efficiencies were assessed by Western Blotting and qPCR (Fig. 4a, b). The results showed successful construction of the MC38^CXCL1−/−^ and MC38^CXCL1+/+^ cell lines. The cell biological behavior was then examined by in vitro assays. In this part, we studied the effect of CXCL1 on the proliferation of colon cancer cells by CCK-8 and clone formation assays. CCK-8 results showed that MC38CXCL1 + / + cells had the fastest proliferation rate, followed by MC38 WT cells, while MC38^CXCL1−/−^ cells had the slowest proliferation rate (Fig. 4c). Similar results were obtained in cell clonal formation experiments (Fig. 4e). In other words, the number of MC38 cells overexpressing CXCL1 was significantly higher than that of wild-type cells, while the number of MC38 cells with CXCL1 knockout was significantly lower than that of wild-type cells. This result suggested that CXCL1 can promote the proliferation of colon cancer cells. We examined the effect of CXCL1 on migration of colon cancer cells using Transwell assay. The results showed that CXCL1 overexpression significantly promoted the migration of colon cancer cells, while CXCL1 knockout significantly inhibited the migration of colon cancer cells (Fig. 4d). These findings suggest that CXCL1 exerts its role as an oncogene to regulate cell proliferation and migration in colon cancer.

### CXCL1 promotes colon cancer growth and angiogenesis

**Fig. 5:**
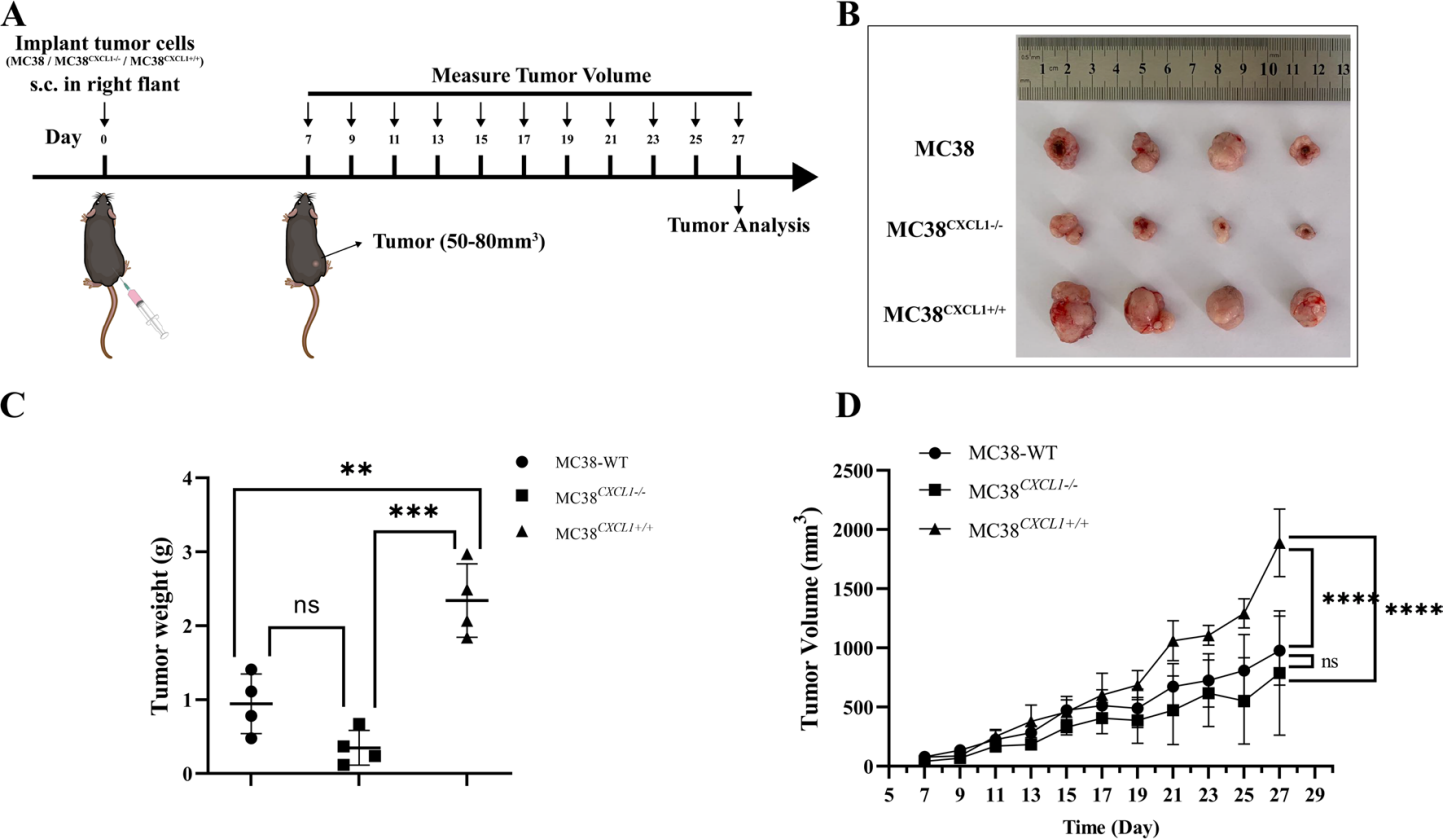
CXCL1 promoted colon cancer development in mouse models. **a** Experimental protocol for measuring the effect of *CXCL1* knockout and overexpression in subcutaneous graft colon cancer models; **b** photographs of the tumors; **c** mean tumor weight; **d** tumor growth curve. Each data point represents mean ± SD (n = 4). *ns* no significant, **P < 0.01, ***P < 0.001, ****P < 0.0001

To further validate the promoting effect of CXCL1 on colon cancer growth in vivo, we transplanted three cell lines (MC38 WT, MC38^CXCL1−/−^, MC38^CXCL1+/+^) percutaneously at the right flank of C57BL/6J male mice and started assessing the tumor volume 1 week later (Fig. 5a). The result showed that overexpression of CXCL1 in colon cancer cells significantly promoted colon cancer growth, while knockout of *CXCL1* in colon cancer cells inhibited colon cancer growth (Fig. 5b–d).

**Fig. 6:**
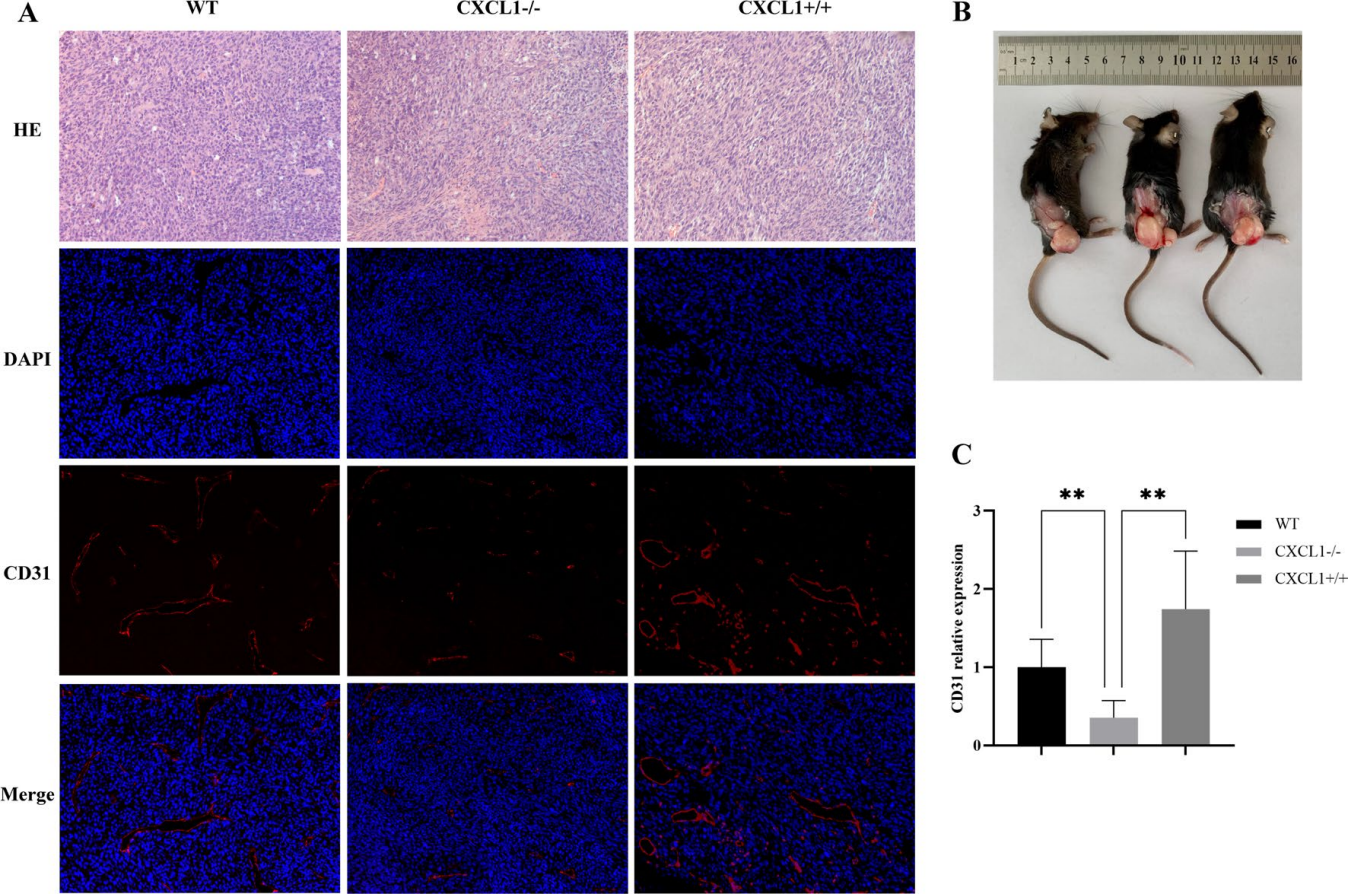
Positive correlation between CXCL1 and the number of tumor vessels. **a, c** Immunofluorescence assay revealed that *CXCL1* overexpression significantly elevated CD31 expression in the colon cancer models, whereas *CXCL1* knockout in MC38 resulted in an inhibition of CD31 expression (× 200). **b** MC38.^CXCL1+/+^ tumors surrounded by abundant blood vessels. **P < 0.01

In addition, dissection of tumor tissues revealed significant vascular infiltration in tumors of mice overexpressing CXCL1 (Fig. 6b). Therefore, we hypothesized that CXCL1 promotes angiogenesis and colon cancer. To verify the relationship between CXCL1 and angiogenesis, we examined CD31 in the tumors of each group of mice using immunofluorescence, and the results showed a significant increase in CD31 expression after CXCL1 overexpression, while the opposite result was observed after CXCL1 knockout (Fig. 6a, c). These data suggest a positive correlation between CXCL1 and CD31 expression. However, further studies are required to identify the pathway by which CXCL1 leads to increased vascularity in colon cancer.

**Fig. 7:**
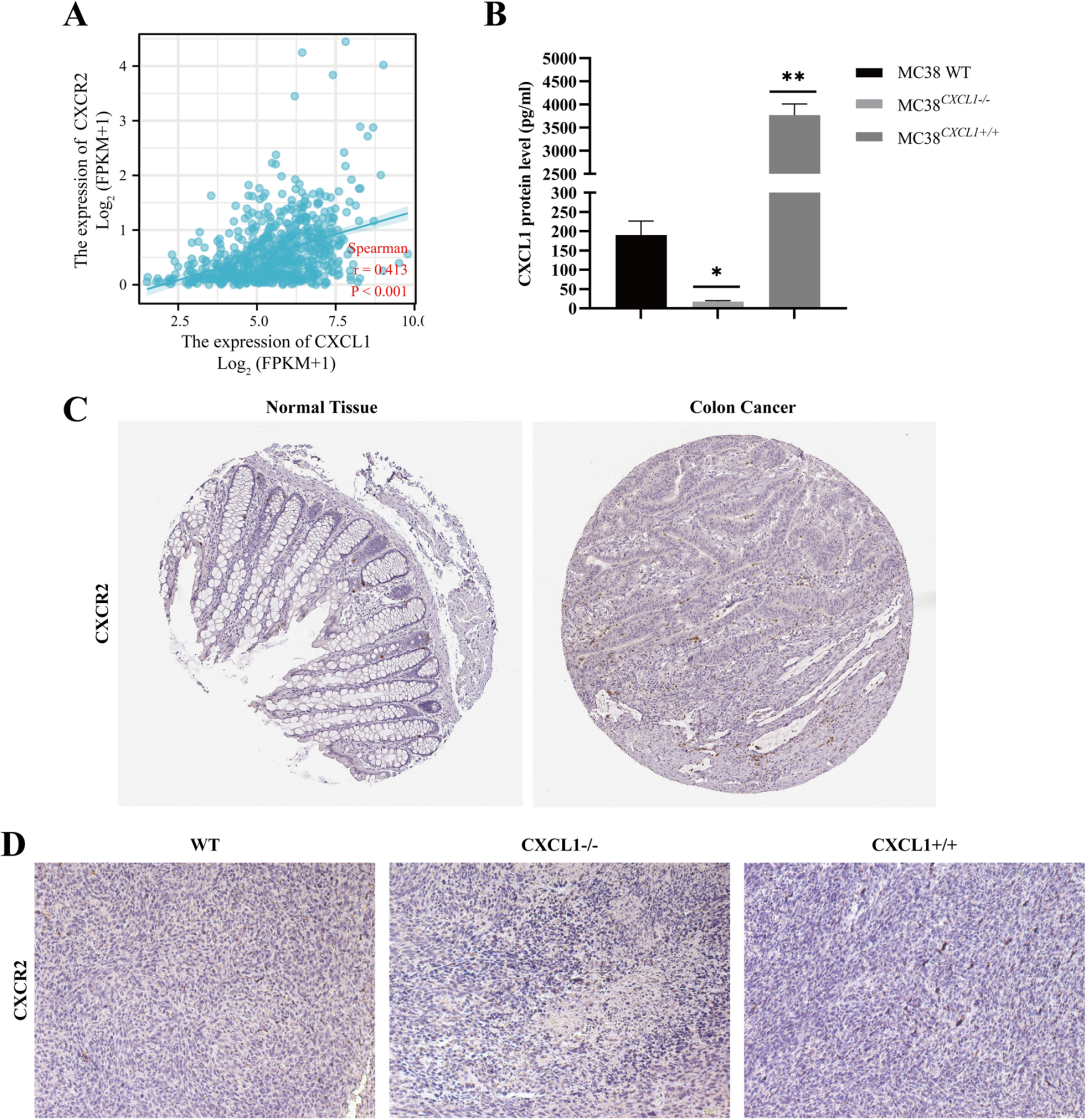
CXCL1 cannot exert its effects through autocrine manner in colon cancer. **a** Results of bioinformatics analysis showing a positive correlation between *CXCL1* and the expression of CXCR2. **b** The protein expression of *CXCL1* in the supernatant derived from each group of cells (MC38 WT, MC38^CXCL1−/−^, MC38^CXCL1+/+^) was assessed by ELISA assay. **c** The CXCR2 protein expression profile in the HPA database. **d** Immunohistochemistry assay analyzed the expression of CXCR2 in mouse colon cancer tissues. *P < 0.05, **P < 0.01

To determine whether the autocrine mechanism is involved in the CXCL1-mediated malignant process in colon cancer, we measured the CXCL1 secretion of each group of cells (MC38 WT, MC38^CXCL1−/−^, MC38^CXCL1+/+^) by ELISA, and the results showed that the CXCL1 protein secreted in CXCL1 knockout cells decreased significantly, while the CXCL1 protein secreted in overexpressing CXCL1 cells was significantly increased (Fig. 7b). The function of CXCL1 is mainly mediated by G protein-coupled receptor CXCR2 binding, and TCGA database analysis shows that there is a high positive correlation between CXCL1 and CXCR2 RNA expression in colorectal cancer (Fig. 7a). Later, we analyzed the expression of CXCR2 in various groups of mouse tumors by immunohistochemistry, and the results showed that CXCR2 was localized on the colon cancer cell membrane and cytoplasm (Fig. 7d), which provided the possibility of binding to CXCL1, but the protein expression level of CXCR2 was negligible compared to the high level of CXCL1 expression in colon cancer cell lines. The results in the HPA database also showed that CXCR2 was almost not expressed in normal colon tissue and colon cancer tumor tissue (Fig. 7c). Based on the above results, we believe that CXCL1 cannot mediate the development of colorectal cancer through autocrine mechanisms.

### CXCL1 expression is correlated with immune cell infiltration in colon cancer

**Fig. 8:**
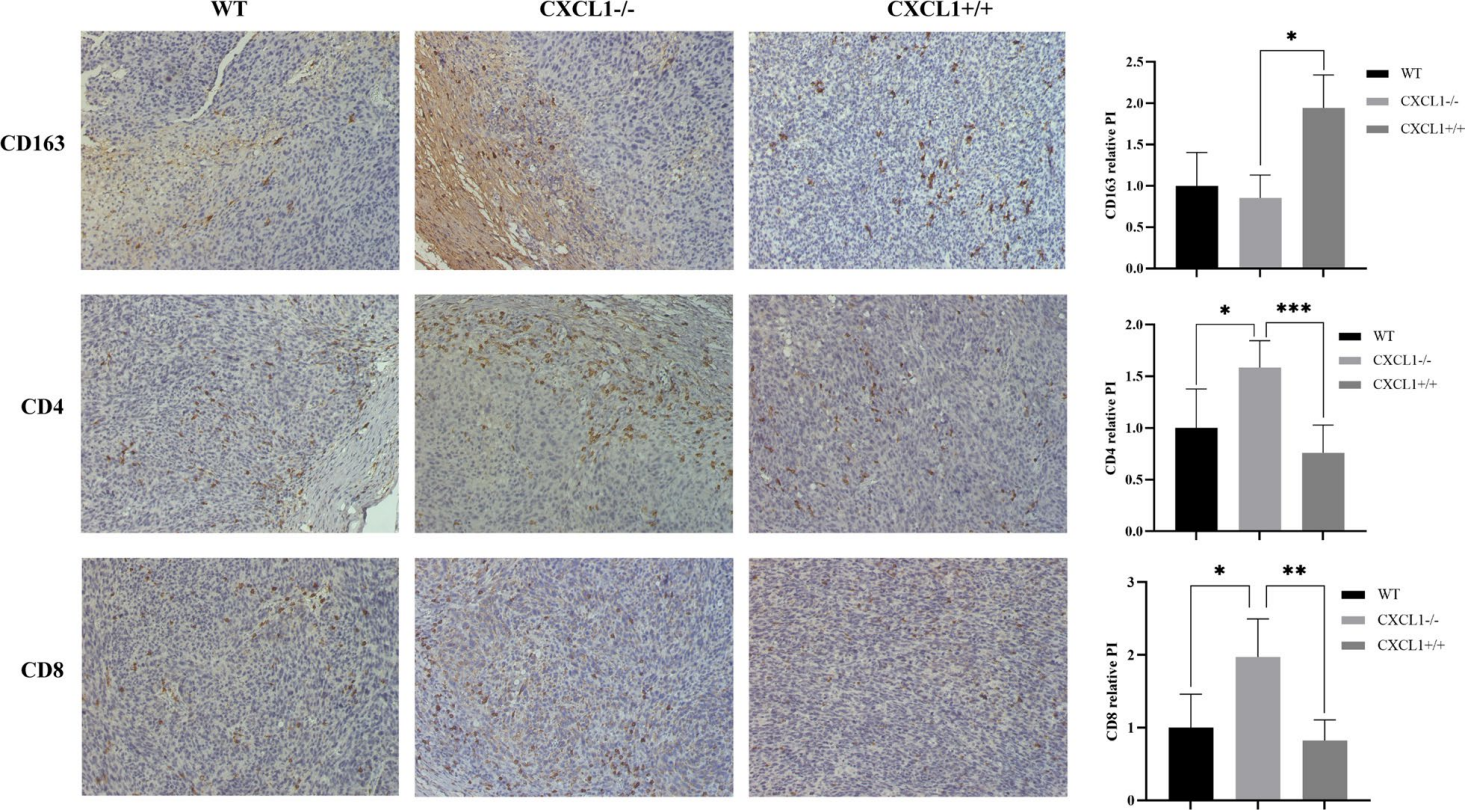
Relationship between CD163, CD4, and CD8 infiltration and expression of CXCL1 in colon tumor tissues. Immunohistochemistry assay revealed that *CXCL1* overexpression significantly elevated CD163 infiltration in colon tumor tissues, whereas *CXCL1* knockout in MC38 led to increased CD4 and CD8 cell infiltration (× 200). *P < 0.05, **P < 0.01, ***P < 0.001

The tumor microenvironment plays an indispensable role in tumorigenesis and tumor progression. The interaction between tumor microenvironment and colon cancer is mainly mediated by multiple immune cells and their secreted cytokines, which affect tumor proliferation and metastasis. To detect the effect of CXCL1 on the tumor microenvironment, we examined CD4, CD8, and CD163 expression in tumor tissues of each group of mice (Fig. 8). The results showed that CXCL1 overexpression in colon cancer cells increased the infiltration of tumor-associated macrophages (TAMs), while decreasing the CD4 and CD8 cells around the tumor. The infiltration of CD4, CD8, and CD163 in tumor tissues after *CXCL1* knockout was not significantly different from that in wild-type colon cancer tissues. These data suggest that CXCL1 overexpression reduces T-cell aggregation and increases infiltration of M2-TAMs in colon cancer tissues.

### CXCL1-mediated colorectal cancer development is NF-κB/P300-dependent

**Fig. 9:**
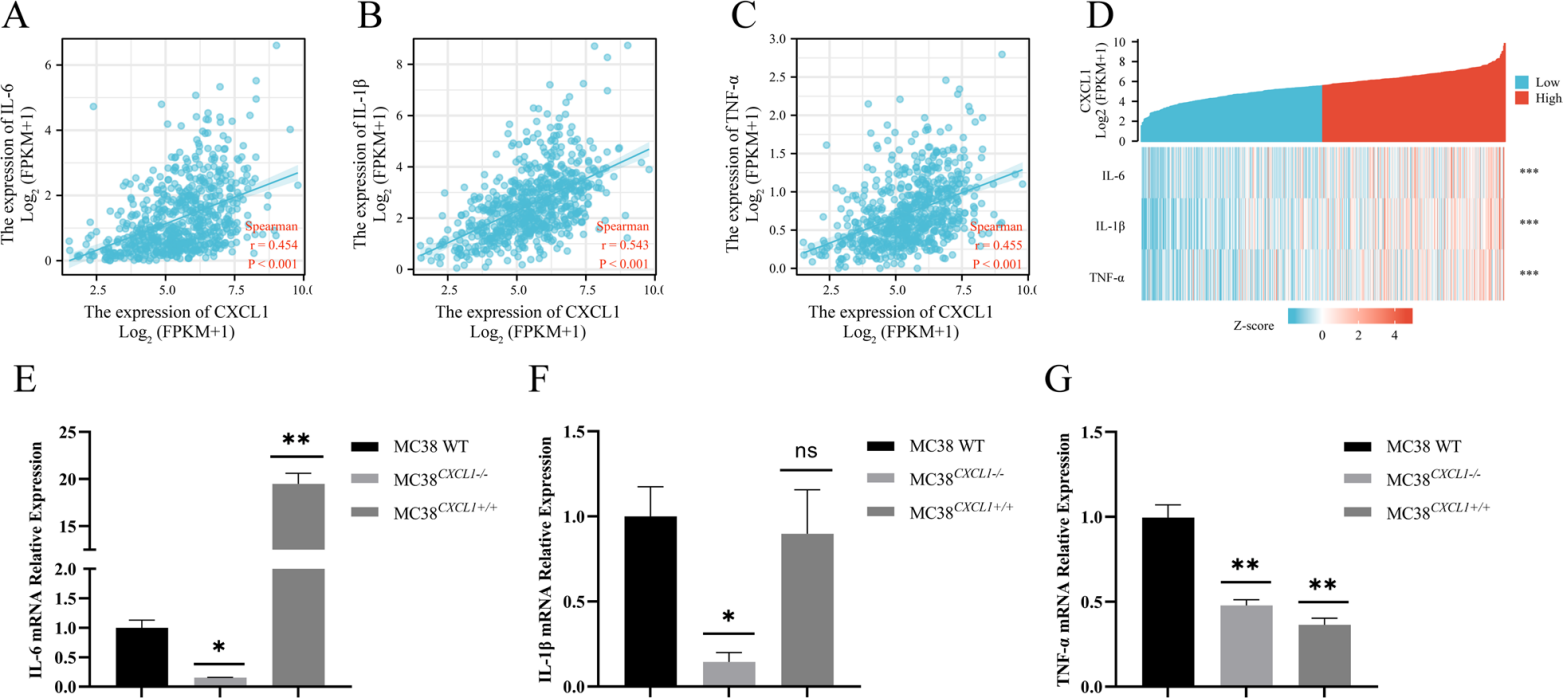
CXCL1 expression is correlated with the activation of NF-κB signaling pathways. **a–d** Results of bioinformatics analysis showing a positive correlation between *CXCL1* and the expression of IL-6, IL-1β, and TNF-α. **e–g** Real-time fluorescence quantitative PCR analysis of IL-6, IL-1β, and TNF-α expression after *CXCL1* knockout and overexpression in MC38 colon cells. *ns* no significant, *P < 0.05, **P < 0.01

CXCL1, one of the most important chemokines, is involved in the development of several inflammatory diseases and shows elevated expression in the inflammatory response [23]. After carcinogen-induced expression, CXCL1 can lead to chronic inflammation by recruiting neutrophils, resulting in tumor formation [23–25]. CXCL1 has been reported to activate NF-κB in other cancer types; however, whether CXCL1 can activate NF-κB in colon cancer has rarely been reported. Therefore, we examined the activity of the NF-κB pathway. Firstly, we analyzed the correlation between CXCL1 and several key genes related to the NF-κB pathway by bioinformatics analyses, and the results showed a significant moderate to strong positive correlation of CXCL1 with IL-6, IL-1β, TNF-α, and IFNγ (Fig. 9a–d). To further verify the results, we examined the expression of these genes in MC38, MC38^CXCL1−/−^, and MC38^CXCL1+/+^ cells by real-time fluorescence quantitative PCR. The results showed that the mRNA expression levels of IL-6 remained consistent with CXCL1 expression levels; IL-1β expression was reduced after CXCL1 knockdown and no significant changes were observed after CXCL1 overexpression; while TNF-α expression was reduced in both (Fig. 9e–g). This result suggested a significant association between CXCL1 and NF-κB pathway.

**Fig. 10:**
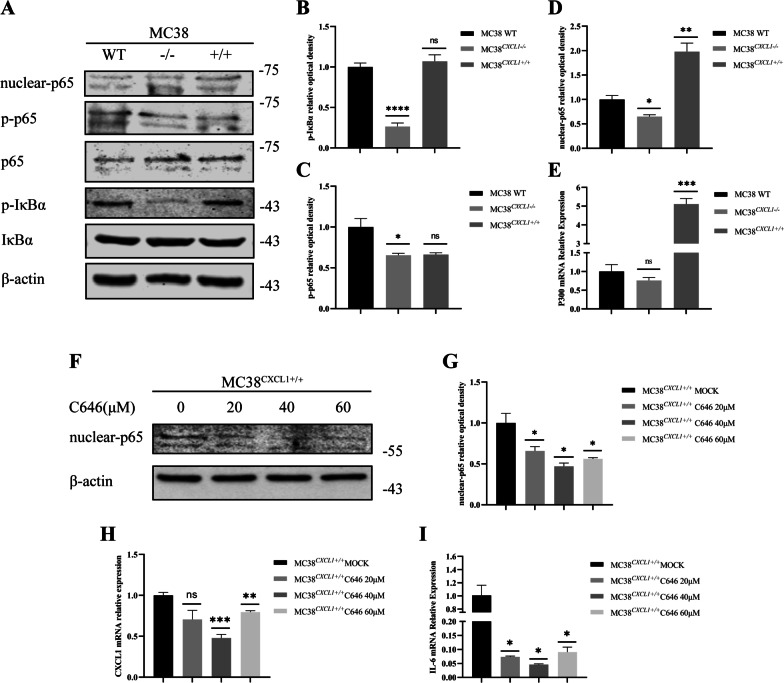
CXCL1 activates the NF-κB pathway via P300. **a–d** *CXCL1* activated NF-κB signaling in MC38 colon cancer cells, presenting as increased expression levels of p-IκBα/IκBα, nuclear-p65/p65. **e** *CXCL1* overexpression in MC38 colon cancer cells promoted P300 mRNA expression. **f, g** C646 treatment significantly inhibited the nuclear-p65 expression. **h, i** mRNA expression levels of *CXCL1* and IL6 decreased after C646 treatment of MC38^CXCL1+/+^ cells, and the decreased level of IL6 remained consistent with *CXCL1*. *ns* no significant, *P < 0.05, **P < 0.01, ***P < 0.001, ****P < 0.0001

Further, we detected the activation of NF-κB pathway by Western Blotting (Fig. 10a–d). The result showed that the phosphorylation level of IκBα was significantly decreased after CXCL1 deletion, while there was no significant change in the phosphorylation level of IκBα after CXCL1 overexpression. However, CXCL1 deletion and overexpression did not significantly affect the phosphorylation of total p65. Since NF-κB needs to enter the nucleus to regulate transcription, we also examined the expression of p65 protein level in the nucleus to assess whether NF-κB was activated. The results showed that CXCL1 overexpression led to a significant increase in the expression of p65 protein level in the nucleus. The above results suggested that CXCL1 is an upstream factor of the NF-κB pathway and its activation of NF-κB pathway may not depend on phosphorylation of p65, whereas NF-κB activation can be achieved by acetylation in addition to phosphorylation.

**Fig. 11:**
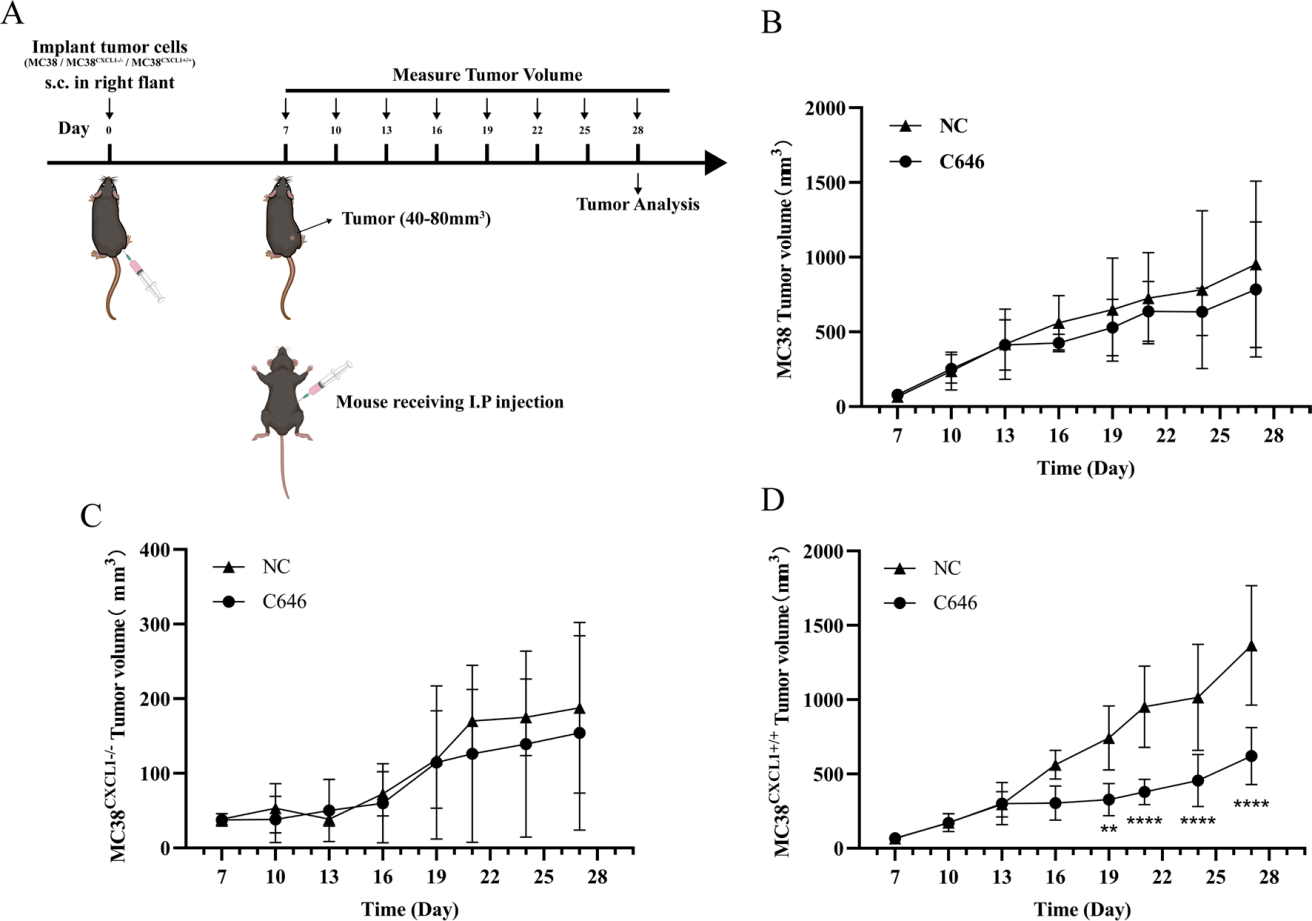
C646 treatment inhibited MC38^CXCL1+/+^ colon cancer tumor progression. **a** Experimental protocol for measuring the effect of C646 treatment in subcutaneous graft colon cancer models. **b–d** Tumor growth curve after treatment with C646 (MC38 WT, MC38^CXCL1−/−^, MC38^CXCL1+/+^). Administered by intraperitoneal injection every three days. Each data point is the mean ± SD (n = 5). **P < 0.01, ****P < 0.0001

**Fig. 12:**
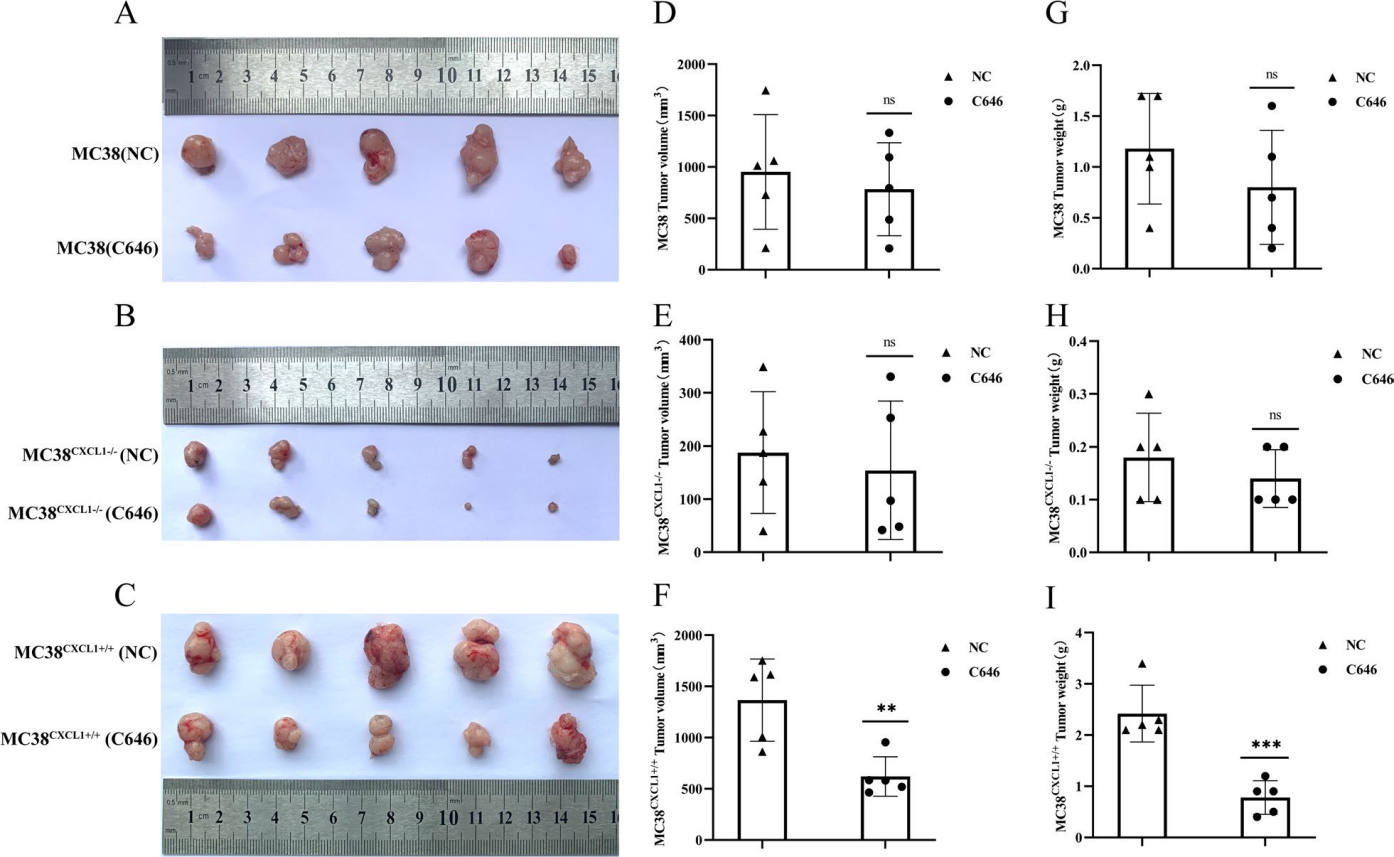
C646 treatment inhibited MC38^CXCL1+/+^ colon cancer tumor progression. **a–c** Photographs of the tumor after treatment with C646 (MC38 WT, MC38^CXCL1−/−^, MC38^CXCL1+/+^). **d–f** Mean tumor volume after treatment with C646 (MC38 WT, MC38^CXCL1−/−^, MC38^CXCL1+/+^). **g–i** Mean tumor weight after treatment with C646 (MC38 WT, MC38^CXCL1−/−^, MC38^CXCL1+/+^). *ns* no significant, **P < 0.01, ***P < 0.001

P300, an important histone acetylase, has been reported to directly acetylate the non-histone transcription factor p65, thereby affecting cell growth and differentiation. To verify the role of P300 in mediating CXCL1 activation of the NF-κB pathway, first, we examined the mRNA expression of P300 in MC38, MC38^CXCL1−/−^, and MC38^CXCL1+/+^ cells by real-time fluorescence quantitative PCR. The results showed significantly increased expression level of P300 in the MC38 cells overexpressing CXCL1 (Fig. 10e). Then, we added the P300 inhibitor C646 to the cell culture system. After adding different doses of C646 to MC38^CXCL1+/+^ cells, C646 inhibited the expression of CXCL1 and IL-6 to different degrees, and the expression trends of CXCL1 and IL-6 remained consistent (Fig. 10h, i). Subsequent Western Blotting results showed significantly decreased expression level of p65 in the nuclei of MC38^CXCL1+/+^ cells treated with C646 (Fig. 10f, g). The above results suggested an inhibitory effect of C646 on the activation of NF-κB pathway. In the animal experiments, we administered C646 intraperitoneally to each group of mice. In the MC38^CXCL1+/+^ group, the tumor volume significantly decreased after C646 treatment, and was restored to MC38 WT level after treatment, while the tumors of mice in the MC38^CXCL1−/−^ group showed no significant changes (Figs. 11, 12). These results suggest a critical role of P300 in mediating CXCL1-induced activation of the NF-κB pathway.

## Discussion

In the present study, we applied bioinformatics analysis and identified increased expression of CXCL1 in colorectal cancer. Previous analyses of clinical colorectal cancer tissue samples have also shown consistent results [16]. Subsequently, we explored the role of CXCL1 in colorectal cancer from the perspective of cell biological behavior. We found that CXCL1 promotes the proliferation, migration, and invasion of colorectal cancer cells. In the subsequent mouse model experiments, CXCL1 was found to promote the progression of colon cancer. These results provide the rationale for further studies on the role of CXCL1 in colorectal cancer.

Angiogenesis is a key factor in carcinogenesis and is regulated by multiple molecular pathways. We observed a positive correlation between CXCL1 and the number of tumor vessels in colon cancer. In colorectal cancer patients, CXCL1 also showed a positive correlation with VEGF expression [26]. It has been shown that primary colorectal cancer cells secrete VEGF-A and stimulate TAMs to produce CXCL1 in primary tumors [17], while in tumors of mice treated with chemotherapy, TAMs accumulate around the blood vessels and promote tumor revascularization and recurrence by releasing VEGF-A [27]. In contrast, our data suggest that CXCL1 overexpression increases the expression of CD31 and the infiltration of TAMs. These findings suggest a close association of CXCL1 with TAMs and tumor angiogenesis. However, further studies are required for in-depth characterization of the underlying mechanism of this association.

In colorectal cancer, there are multiple tumor microenvironments, and the complex interactions between tumors and their microenvironments influence the development of tumors and the metastatic spread of cancer cells [28]. T cell infiltration in tumor tissue is required for tumor regression [29, 30]. Studies have shown that CXCL1 tends to be highly expressed in tumor cells with low T cell clones, that CXCL1 production promotes the recruitment of MDSCs into the tumor, thereby inhibiting CD8 + T cell infiltration, and that CXCL1 production by tumor cells is required for an immunosuppressive phenotype [31]. Studies have also shown that RIP3 increases CXCL1 expression thereby promoting myeloid cell-induced adaptive immunosuppression in tumors [32, 33]. In addition, CXCL1 produced in primary colorectal cancer cells was shown to form a pre-migratory ecology by recruiting MDSCs to eventually promote liver metastasis [17]. In contrast, the CXCL1 receptor CXCR2 antagonist, SB225002, was found to reduce the accumulation of PMN-MDSCs and increase CD8+ T cell infiltration in tumors [34]. Our bioinformatics results suggested an association between CXCL1 and multiple immune cells, while overexpression of CXCL1 in a subsequent mouse model showed increased infiltration of CD4+ and CD8+ T cells. These findings suggested that CXCL1 increases infiltration of T cells, causes myeloid cell-mediated immunosuppression, and thus promotes tumor progression.

A variety of inducible transcription factors, including NF-κB, can be activated by interaction with cellular coactivators [35]. The transcriptional activity of NF-κB can be optimized by interaction with P300/cyclic adenosine monophosphate response element binding protein (CBP) [36]. P300/CBP, a transcriptional co-activator, is believed to regulate transcription through its histone acetyltransferase (HAT) activity [37, 38]. Through enzymatic activity localized to the HAT structural domain, P300/CBP activates transcription by transferring acetyl groups to the ε-amino groups of histone lysine residues, leading to elevated levels of histone 3 lysine 27 acetylation (H3K27ac), P53 [39, 40], and NF-κB acetylation, thereby promoting expression of a variety of genes. In the present study, CXCL1 was found to activate the NF-κB pathway in colon cancer cells, which is consistent with previous reports. In the study by Kou et al., CXCL1 increased nuclear translocation of NF-κB and activated the NF-κB/HDAC1 pathway to promote progression of prostate cancer [41]. In contrast, NF-κB-mediated CXCL1 production was found to contribute to the maintenance of bone cancer pain, suggesting some regulatory relationship between NF-κB and CXCL1. In the present study, treatment of CXCL1-overexpressing colon cancer cells with C646 (an inhibitor of P300) led to inhibition of CXCL1 expression along with decrease in the protein level of p65 in the nucleus. Knockout of *CXCL1* in colon cancer cells significantly inhibited the growth of tumors in mice compared to that in the other groups. Upon subsequent treatment of mice with C646, the tumors could not be further reduced. In contrast, C646 treatment of mice with CXCL1-overexpressing colon cancer significantly inhibited tumor growth, suggesting the involvement of P300 in CXCL1-mediated bioactivity.

In conclusion, we demonstrated overexpression of CXCL1 in colon cancer tumors. CXCL1, an important cytokine that promotes colon cancer development, induces myeloid cell-mediated immunosuppression. Inhibition of P300 activity blocked NF-κB activation and CXCL1-induced pro-tumor growth effects. Our findings suggest the involvement of NF-κB/P300 in CXCL1 downstream signaling. Therefore, CXCL1 is a potential prognostic biomarker and therapeutic target in the context of colorectal cancer.

## Data Availability

TCGA-COAD dataset containing RNA-seq (FPKM) and clinical information was downloaded from The Cancer Genome Atlas (TCGA) database in March 22, 2022. GSE113513, GSE25070, GSE41328, and GSE156355 dataset was obtained from Gene Expression Omnibus (GEO) database in March 22, 2022.
